# Application of Machine Learning in Translational Medicine: Current Status and Future Opportunities

**DOI:** 10.1208/s12248-021-00593-x

**Published:** 2021-05-18

**Authors:** Nadia Terranova, Karthik Venkatakrishnan, Lisa J. Benincosa

**Affiliations:** 1 Translational Medicine, Merck Institute for Pharmacometrics, Merck Serono S.A., Lausanne, Switzerland; 2 Translational Medicine, EMD Serono Research & Development Institute, Inc., Billerica, Massachusetts USA

**Keywords:** digital innovation, machine learning, model-informed drug discovery and development, pharmaceutical R&D, Translational Medicine

## Abstract

The exponential increase in our ability to harness multi-dimensional biological and clinical data from experimental to real-world settings has transformed pharmaceutical research and development in recent years, with increasing applications of artificial intelligence (AI) and machine learning (ML). Patient-centered iterative forward and reverse translation is at the heart of precision medicine discovery and development across the continuum from target validation to optimization of pharmacotherapy. Integration of advanced analytics into the practice of Translational Medicine is now a fundamental enabler to fully exploit information contained in diverse sources of big data sets such as “omics” data, as illustrated by deep characterizations of the genome, transcriptome, proteome, metabolome, microbiome, and exposome. In this commentary, we provide an overview of ML applications in drug discovery and development, aligned with the three strategic pillars of Translational Medicine (target, patient, dose) and offer perspectives on their potential to transform the science and practice of the discipline. Opportunities for integrating ML approaches into the discipline of Pharmacometrics are discussed and will revolutionize the practice of model-informed drug discovery and development. Finally, we posit that joint efforts of Clinical Pharmacology, Bioinformatics, and Biomarker Technology experts are vital in cross-functional team settings to realize the promise of AI/ML-enabled Translational and Precision Medicine.

## INTRODUCTION

Big data and technological innovation have revolutionized medicine and healthcare over the last decade (1). Today, advanced technological solutions are able to generate health and medical data at the individual level in real time and in a real-world environment. They are at the core of such digital disruption that holds promise for improving the practice of medicine towards a more targeted and personalized paradigm, enabled by data-driven decisions based on real-world evidence, patient-participatory drug development, and healthcare democratization. In recent years, pharmaceutical Research and Development (R&D) has transformed to a highly dynamic process enabled by patient-centered iterative forward and reverse translation. Traversing the path from idea to medicine has become increasingly multi-disciplinary and inter-connected, as exemplified by the Drug Discovery, Development, and Deployment Map, illustrating a network view of the process and associated cross-sector ecosystem, challenging the typical chevron (linear, sequential, left to right) view of pharmaceutical R&D pipeline (2). A Bayesian learning mindset that exploits the totality of evidence is particularly important in timely delivery of innovative healthcare solutions to address unmet medical needs with the right sense of urgency (3–5).

Whereas advances in biology, biomedical engineering, and computational sciences have resulted in an explosive increase in our ability to generate and store multi-dimensional data from diverse sources (e.g., laboratory, clinical trial, real-world, literature), consistent real-time integration of these data for principled and timely decision making in pharmaceutical R&D and healthcare remains aspirational (6). Recognizing this critical importance of optimal knowledge management, the pharmaceutical industry has started building digital capabilities and embracing innovations in Data Science into their Research and Development (R&D) organizations (7). Machine learning (ML), deep learning (DL) and more generally artificial intelligence (AI) techniques are central components of this innovation. While AI refers to the output of a computer generated by mimicking a human behavior and it does not say how the problem has been solved, ML is a subset of AI consisting of a set of algorithms that parse data, learn from them, and then apply learnings to make intelligent decisions. A simple and widely used classification of ML algorithms is into supervised, unsupervised, DL and then reinforcement learning. Supervised learning is task driven and it is used for classification and prediction tasks on new data by starting on datasets with known labels or outcomes. Differently, unsupervised learning methods are data driven and focus on finding structures and patterns inside the data itself. Examples include finding groups and clusters (clustering), understanding relationships between items (association rule mining), and finding a more compact representation of the data (dimension reduction). Reinforcement learning uses algorithms interactively learning to react to an environment from mistakes by focusing on decision and policy making. Lastly, inspired by the biological neural network (NN) of the human brain, DL uses NNs with many layers (more than one hidden layer to be considered deep) to solve the hardest (for computers) problems. Such process of learning is far more capable than that of standard ML models. Indeed, while both ML and DL fall under the broad category of AI, DL is what powers the most human-like artificial intelligence.

Owing to their ability to learn hidden and predictive patterns in large amounts of heterogenous and high-dimensional datasets, AI techniques have been increasingly adopted across the drug discovery and development value chain (8). Initially, AI methods were mostly used in drug discovery to analyze large sets of chemical structure data, gene expression and genetic data, and high throughput *in vitro* data (9). Optimization of drug candidates towards better drug properties has been an area of sustained focus of AI/ML frameworks in drug discovery, enabling efficient iterative approaches to multi-dimensional optimization based on virtual screening and prediction of physicochemical properties and biological activity and toxicity (9). More recently, a wide range of ML applications have emerged as promising approaches to generate new knowledge in translational and clinical drug development (10). Together with the automation of process pipelines and operational design, the integration and analysis of large, multi-dimensional, and heterogenous data sets, such as -omics data, information from wearable devices, images, and electronic health records, offers an unprecedented opportunity for AI/ML applications in drug development. The associated contexts of use range broadly from advancing understanding of the disease and its underlying physiological and biological underpinnings, elucidation of drug mechanism of action (MoA) and identification of promising combinations, characterization of sources of population variability in patients’ response, and enhancement of trial design and operational efficiency, diagnosis, individualized treatment, and precision dosing solutions (11–13).

**Fig. 1:**
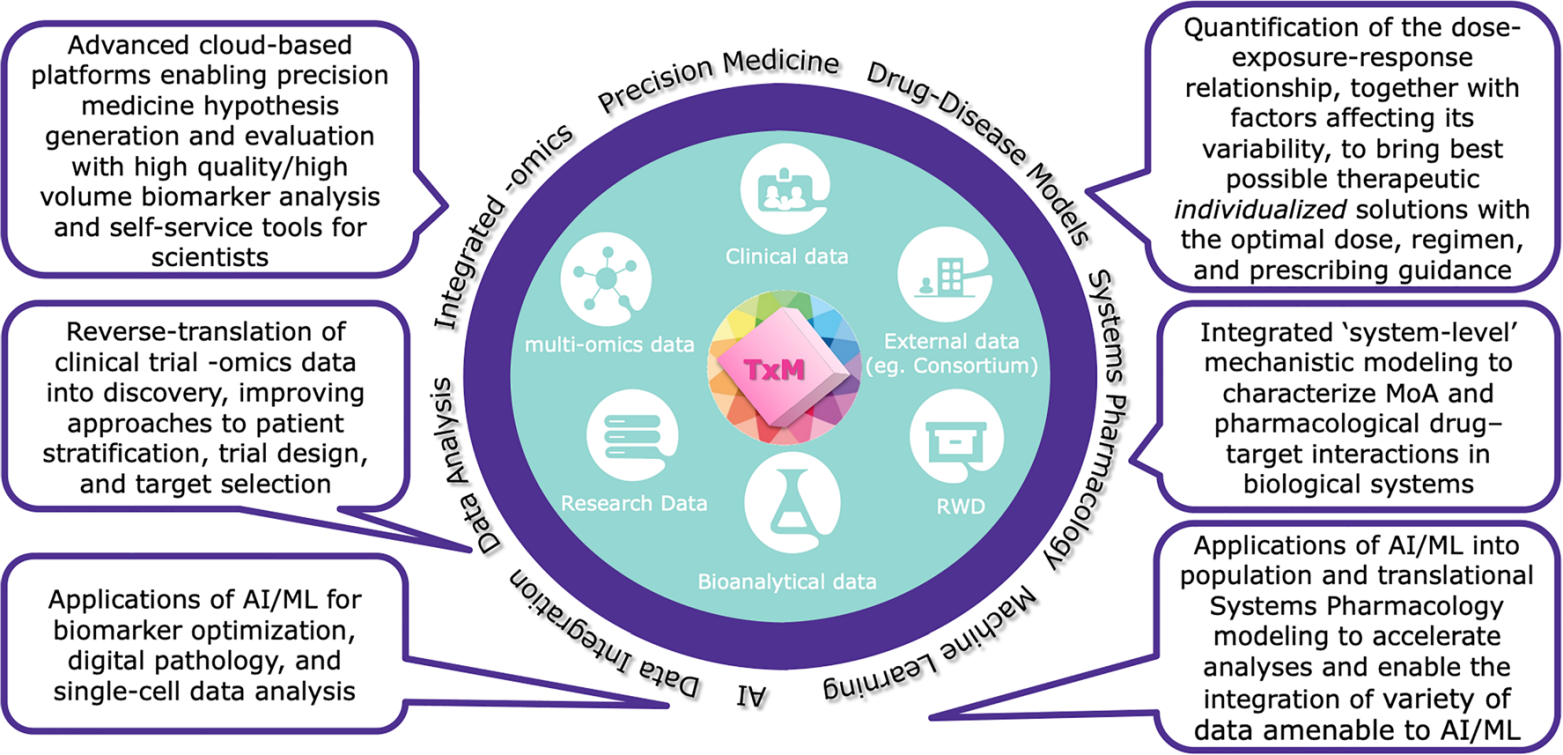
Advanced analytics as a strategic enabler of Translational Medicine. With multiple data sources at its center, Translational Medicine relies on quantitative integration powered by multiple advanced analytical solutions to innovatively support forward and reverse translation with a strategic focus on building confidence in target, patient, and dose

The integration and use of AI/ML methods across the translational through clinical drug development continuum have already demonstrated a clear impact on our ability to successfully maximize the value of data. Furthermore, these methods have enhanced knowledge management both with respect to the studied drug and the disease/patient population, thereby enabling optimization of R&D across the three key inter-dependent strategic pillars that constitute the practice of Translational Medicine: *target*, *patient*, and *dose* (14). These pillars represent the fundamental pivots for hypothesis generation and ultimately for data-driven knowledge generation from preclinical, clinical, and regulatory evaluations designed to build a body of scientific evidence to achieve clinical proof of concept of innovative investigational therapies. Robust and efficient data-driven optimization alongside these three pillars is crucial to maximize probability of success in clinical development and ultimately to successfully impact product registration, labeling, and guidance for therapeutic use at the right dosage and in the context of applicable personalized medicine strategies in concert with companion diagnostics where relevant, to maximize benefit/risk across populations and clinical contexts of use. A quantitative mindset that collaboratively synergizes the disciplines of biomarker sciences, pharmacometrics, systems pharmacology, and bioinformatics is vitally important to the successful practice of Translational Medicine (15). Accordingly, advanced analytics represents a key enabler for Translational Medicine to innovatively support forward and reverse translation (Fig. 1).

In this commentary, we provide an overview of ML applications in drug discovery and development, aligned with the three strategic pillars of Translational Medicine (target, patient, dose) and offer perspectives on their potential to transform the science and practice of the discipline.

## TARGET

The objective of the “target” strategic pillar is to identify the right biological target for a selected disease. Confidence in the biological target and therapeutic hypothesis must be built to initiate the discovery of drugs modulating this target and consequently the disease. This process, also known as target selection and validation, is data driven. It uses a wide range of experiments and multi-dimensional datasets that can inform the identification and selection of novel targets and provide evidence of their association with a disease. Various ML applications using computational druggability prediction methods have emerged for prioritizing target selection by reducing the potential space of druggable targets, and for elucidating the target-disease causality (8). These include the prediction of druggable genes at the genome-wide scale by constructing, for example, a decision tree-based meta-classifier and training it on data including network topological features, tissue expression profile, and subcellular localization (16). Use cases accommodate predictions for specific cancer types based on a variety of genomic and systemic datasets fed into a support vector machine (SVM) classifier and then target validation through inhibition with antibodies, synthetic peptides, and small molecules (17). Supervised ML classifiers predicting whether a small-molecule drug can be generated for any given target have also been generated by integrating rich data including physicochemical, structural, and geometric attributes (18). Emerging and novel therapeutic modalities (e.g., effectors of protein-protein interactions, nucleic acid therapeutics) will demand unique considerations (19). Of relevance for improved patient stratification, Iorio *et al.* identified oncogenic alterations in tumors and found associations with drug sensitivity/resistance, thus highlighting the importance of tissue lineage in mediating drug response (20). Significant work has been done for designing and screening effective drug combinations which are commonly used to treat patients with complex diseases that respond poorly to single-agent therapies. For example, a novel network propagation-based method with Random Forest (RF) models predicting anticancer drug synergy to the accuracy of experimental replicates has been established by integrating the cross-cell and cross-drug information from a large drug combination screening dataset and assembling the information in monotherapy and simulated molecular data (21). Recent advances in natural language processing (NLP) have further unlocked information and knowledge on targets and target-disease association present in literature data by enabling effective and efficient access to available and unstructured sources (22). These methods can additionally be deployed for elucidating the molecular basis of drug-related toxicities resulting from off-target interactions, thereby informing hypothesis generation for next-generation (“best-in-class”) drug design aimed at maximizing therapeutic index (23).

A key component in target validation is building of confidence in the therapeutic hypothesis using quantitative systems pharmacology (QSP) models. Such mechanistic models integrate information on drug pharmacokinetics (PK), target binding, and biological processes of interest and mechanisms of action, resulting from prior knowledge and available preclinical and clinical data, to quantitatively predict efficacy and safety responses over time and translate molecular data to clinical outcomes (24). QSP provides an ideal quantitative framework for integration of diverse big data sources, including omics (i.e., genomics, transcriptomics, proteomics, and metabolomics) and imaging, the dimensionality of which can be reduced by using ML methods. By allowing the identification of relevant association and data representations, the development of QSP platforms with higher granularity and enhanced predictive power can be further enhanced (25). For example, Ramm *et al.* used systems biology approaches combined with multi-dimensional datasets and ML to identify biomarkers predicting nephrotoxic compounds and better characterize their mechanism of toxicity *in vitro* (26). Specifically, an RF algorithm was used for the systematic identification of imaging features and genes best distinguishing between kidney toxic and nontoxic compounds, and hierarchical clustering allowed to identify compounds with similar mechanism of action. Given the complex, typically nonlinear and computationally intensive nature of these models, the use of ML methods has also been envisaged for improving the efficiency of parameter sensitivity and model identifiability analyses (27).

After correctly identifying the target of interest, the next step in drug discovery and development is to design active compound(s) that can produce the intended effect(s) on the targeted gene or protein. Examples of drug screening approaches exploiting ML features propose the use of DL to enable ligand-based virtual screening (28) or to design compounds with almost optimal values for solubility, PK properties, bioactivity, and other parameters through reinforcement learning (29, 30). Deep NNs (DNNs) have been also employed for understanding molecular structure (31) through structure-based protein analysis (32) and prediction of drug-drug interactions (33). Recently, there has been an increasing trend to apply methods similar to quantitative structure-activity relationship (QSAR) in protein design and engineering for biologics therapeutics development (34).

The identification of favorable drug repositioning opportunities offers a rich data ground for the application of state-of-the-art ML methods, including DNNs, SVMs, elastic net regression, RF, and gradient boosted trees, leveraging variety of data sources like gene expression data, molecular pathways, drug binding affinities, and clinical trials data, to identify existing approved drugs having adequacy for the treatment of a never-considered therapeutic indication. Some efforts in this direction focused on applications for repurposing drugs for schizophrenia as well depression and anxiety disorders (35). In this context, the potential value of AI-based deep learning models integrated into proprietary and big data platforms has also been recently discussed to predict drug structures active against SARS-CoV-2 to treat COVID-19 (36).

## PATIENT

The “patient” pillar underscores the importance of selecting the right patient population for the investigational drug and identifying the potential use of a companion diagnostic test. The goal is to identify the right patient group in the selected disease. Although selection of the right patient population for many successful precision therapeutics has been informed by alterations in specific genes (e.g., mutations in the epidermal growth factor receptor in non-small cell lung cancer dictating selection of an appropriate molecularly targeted kinase inhibitor), current problems in precision medicine are far more complex where the impact of large-scale pharmacogenomic variation on drug response is interrogated, made possible through advances in next-generation sequencing and bioinformatics methodologies. Furthermore, deep molecular and clinical characterization of patients results in a multi-dimensional vector of patient characteristics (e.g., genome, epigenome, transcriptome, proteome, metabolome, radiome, and exposome), necessitating ML-based analytical strategies to identify clinically significant signatures of drug effects.

ML-based models have been employed to better understand the MoA of a drug as well as optimizing the definition of patient subgroups. In this context, the identification of biomarkers best predicting efficacy and adverse events of investigational drugs boasts a number of successful use cases ranging from the assessment of translational predictive biomarkers (37) to multi-omics biomarker signatures (38, 39) through methods spanning from partial least-squares regression to RF classifiers. The identification and characterization of patient subgroups with different benefit-risk profiles (e.g., super responders, long-term survivors, non-responders with earlier disease reactivation) is a field that can extensively benefit from the integration of ML applications. For example, the complex determinants of PK and efficacy of cancer immunotherapy have been described extensively for checkpoint inhibitors where increased baseline clearance of protein therapeutics, likely explained by patient-specific disease status factors (e.g., cancer-related cachexia), has been associated with poor clinical outcomes (40). A recent ML-based analysis using RF was able to identify a cytokine signature that was predictive of high baseline clearance of nivolumab and offered generalizable translatability as a predictor of overall survival, representing an example of reverse translation that was able to discover molecular determinants of important associations between baseline (patho)physiology and disease trajectory that have confounded exposure-response understanding for this class of biotherapeutics (41). Importantly, in addition to enabling such associations between patients’ response, or late clinical endpoint, and patients’ baseline factors (including demographics, clinical, genetic, laboratory, and imaging data) or early biomarkers, such algorithms offer the opportunity to integrate and assess the predictive power of large sets of longitudinal factors on the considered outcome. Results from such analyses can then be used to enhance model-informed drug discovery and development (MID3) approaches (42) and advanced disease modeling. For example, radiomics extracts and mines large numbers of medical imaging features quantifying tumor phenotypic characteristics (43) and robust ML methods (e.g., adopting RF) are built to identify reliable quantitative radiomics biomarkers predictive of response (44) and treatment-induced changes in radiomics features. Such signatures are often based on baseline features or early changes. However, as technology advances and costs decrease, extractions of radiomics features from patients’ imaging assessments will become possible beyond early on-treatment time points, thus making available longitudinal tumor makeup features. These can be used to inform more mechanistic pharmacometric (PMX) models of tumor size dynamics accounting for tumor inter- and intra-lesion heterogeneity (45, 46) and resistance emergence (47) after being screened with ML methods or by directly investigating their relationship with model parameters. Similarly, longitudinal patient-level data on each specific genetic alteration quantified by liquid biopsies (LBx) (48) has great potential to be incorporated into population modeling frameworks and enhance our knowledge of molecular disease trajectory and drug MoA as well as determinants of drug resistance, thereby enabling hypothesis generation for combination therapies. By providing a quicker and non-invasive alternative to tissue-based testing, LBx data can be efficiently collected along the patient’s timeline and used to circumvent tumor heterogeneity. This has already been demonstrated by considering aggregated baseline circulating DNA data (49). Looking into the future, we posit that the ability to characterize changes in molecular tumor dynamics over time using LBx data and integrate them in disease modeling can also have a big impact on our understanding of clonal evolution and resistance development. Secondly, it can influence the monitoring of escape mutations to inform treatment sequencing or re-challenge. Finally, optimal design of LBx sampling over time can also be informed by such integrated modeling approaches. In fact, ML-based disease models can be used as a drug development tool to improve trial designs, randomization aids, patient enrichment, and virtual control arms (50). As one example, in the ACCORD trial, random survival forests (RSFs) and a gradient forest analysis allowed the identification of predictors of all-cause mortality in diabetic patients from the analysis of a large number of variables (51, 52). ML-assisted covariate analyses in longitudinal disease trajectory models can help increase confidence in patient selection or stratification strategies. They can provide supportive evidence for predictive biomarkers and inform probability of success for selected *versus* unselected trial designs through Bayesian clinical trial simulations. Of note, recent innovations in disease trajectory modeling have enabled structuring of these longitudinal models in a manner that allows analysis of temporally fragmented population data, as has been applied to describe disease progression in Alzheimer’s disease over a 20-year time period (53). In this context, incorporation of ML represents an untapped opportunity for multi-dimensional covariate analyses on real-world datasets for elucidation of determinants of natural history of disease progression — a key enabler for rare disease drug development. In one application to the anti-diabetic drug metformin, Goswami *et al.* combined a semi-mechanistic disease progression model describing the long-term dynamics of HbA1c following metformin initiation with computational genetic methodologies for elucidation of the clinical and genomic sources of variability in time course of glycemic control (54). By using the hyperLASSO ML technique, the top 9 variants (from 12,000 variants in a selected set of 267 genes) were identified and entered stage 2 of the covariate analysis that employed a pharmacostatistical approach to define the top 3 genetic variants whose contributions to disease progression trajectory were evaluated using clinical trial simulations. Innovations of this nature at the intersection of mechanism-based pharmacodynamic modeling, PMX disease progression modeling, and ML truly represent exemplars for continued progress in our journey toward maximizing knowledge of sources of variability in drug response across global patient populations to optimize pharmacotherapy for all patients through selection of the right drug/combination(s) at the right dose and dosing regimen, which may be different for patients with different genetic backgrounds depending on the mechanism of action of the drug.

Furthermore, as has been discussed in the setting of cancer immunotherapy, personalized disease trajectory models have the potential to bridge to therapeutics as they can enable response-adaptive treatment decisions for individual patients through iterative development and validation of patient-specific models using baseline and on-treatment biomarker and response data collected longitudinally during treatment (55). Given the multi-dimensional nature of such biomarker measurements in oncology as well as other diseases, convergence and synergy across methodologies ranging from mechanism-based (e.g., QSP) models to biologically agnostic (e.g., ML) models will be necessary (56).

Technological advances in image processing and the increased volume of person-generated health data (PGHD) generated by sensors and smart health-tracking devices are playing a key role in improving diagnosis and treatment, and then precision medicine. As an example, Chen *et al.* have shown how longitudinal monitoring of symptoms with several smart devices can be mined for physiological and behavioral signatures of cognitive impairment (CI) by using Extreme Gradient Boosting explained by SHapley Additive exPlanations (SHAP). Particularly, they can provide new avenues for detecting CI in a timely and cost-effective manner and accelerating the development and testing of new therapies (57). The use of DL has greatly influenced modern computer vision. This has led to significant advances in image classifications and object detection in medical imaging specialties such as dermatology, radiology, ophthalmology, and pathology (1). The use of convolutional NNs for the automated detection and quantification of disease biomarkers (58), cancer subtype or gene mutations (59, 60), and distinctive facial syndrome-related phenotypes (61), with accuracy comparable to physicians, holds promise to assist physicians in the generation of more consistent diagnoses. Decision-support applications have the potential to improve reproducibility and treatment decision making. Currently, the most compelling opportunities for AI in precision medicine are offered by big digital pathology data where DL methods have shown superiority in detecting cells and tissues, and image features that may not be visually discernible by a pathologist (62). Combining such data with -omics measures can significantly support better quantitative modeling of disease appearance and evolution, and prediction of patient outcome.

## DOSE

The “dose” pillar of Translational Medicine focuses on selection of the optimal dose and dosing regimen for a drug via an appropriate route of administration and/or drug delivery system across patient populations and clinical contexts of use. It aims identifying the right molecule that delivers the right exposure at the target site of action and elicits the desired target modulation over the stated time period, without compromising patient safety. This translates into building confidence in the therapeutic window based on quantitative characterization of relationships between systemic exposure and the efficacy and safety profile of the studied drug, not only for the typical patient but also across clinical contexts of use in subpopulations. While the discipline of clinical pharmacology has pivoted to this primary purpose of dose optimization across clinical contexts of use in relation to intrinsic and extrinsic sources of variability in drug exposure and response, with increasing complexity in biological mechanisms of action of drugs as well as multi-dimensional sources of biological variability in drug response as discussed earlier, a major opportunity exists for ML-based enhancement of dose optimization strategies. ML-based approaches can help seamlessly bridge knowledge across the biologically rich domain of quantitative systems pharmacology and the pharmaco-statistically oriented domain of pharmacometrics by offering advanced analytical frameworks for iterative forward and reverse translational analysis. The ultimate question for such integrative models is to define the optimal dose, dosing schedule, combination partner, and sequence of administration conditioned on patient-specific factors including biological signatures of (patho)physiology.

There is substantial interest in combining preclinical and clinical data for a better prediction of a compound toxicity. For example, several studies have reported relevant progress in ML-based models for the prediction of drug-induced liver injury (DILI). These studies cover aspects that range from the use of the pattern recognition algorithm decision forest based on structural and molecular data of a large set of FDA-approved drugs (63) to Bayesian ML frameworks mechanistically integrating relevant hepatic safety assays and physicochemical and exposure variables from a chemically diverse compound set (64). By enabling confidential and non-confidential proprietary data exchange across the pharmaceutical industry and institutions, the European Union’s Innovative Medicines Initiative 2 Joint Undertaking (IMI 2) “Enhancing TRANslational SAFEty Assessment through Integrative Knowledge Management (eTRANSAFE)” project (Grant agreement 777365) is aiming at developing an integrative data infrastructure and flexible ML framework allowing for the optimal exploitation of chemical and biological information and translational measures to infer the toxicity of new compounds.

**Fig. 2:**
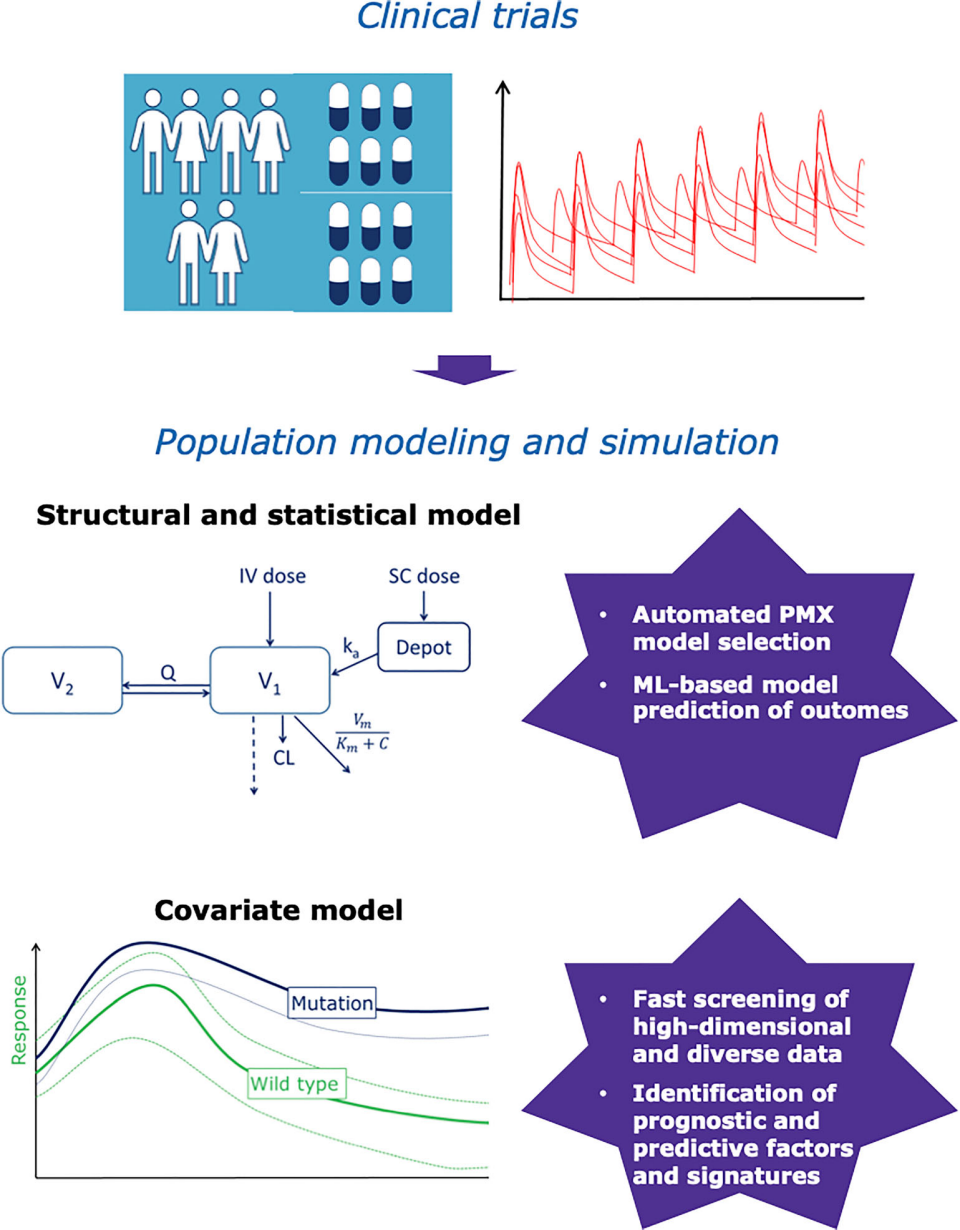
Examples of machine learning applications in the Pharmacometrics model building pipeline. Applications of machine learning are emerging in the Pharmacometrics field. Examples support key model building steps by ranging from the automation and optimization of model selection to the fast and efficient optimization of prognostic and predictive factors from large high-dimensional and diverse datasets. However, there are still several unexplored opportunities present to date to capitalize the full potential of these methods towards next-generation Pharmacometrics

To appropriately inform the therapeutic window, exposure-response relationships for both efficacy and safety must be established. MID3 approaches play a key role in advancing such understanding. These approaches use population PK-pharmacodynamics (PK-PD) models for PD biomarkers, target engagement and drug toxicity, and longitudinal models of disease endpoints (e.g., tumor size dynamics, disease status scales) as well as time to event models of clinical endpoints. One of the key outcomes of such analyses is the assessment of the variability of patient response to the candidate drug and then identification of prognostic and predictive factors. This step has been always limited to predefined and limited sets of variables to reduce model complexity and computational costs. However, ML approaches including RF, NN, and SVM now offer a powerful framework for quickly and efficiently screening large sets of covariates and identifying those relevant to be further explored in a more mechanistic and biologically sound population modeling framework (65). By using the multivariate adaptive regression splines ML method, Hall *et al.* were able to identify the influence of weight and/or age on the PK of dapsone, not identifiable with standard population PK approaches as occurring only between certain ranges of patients’ covariates, delimited by multiple regions of discontinuity (66). In another example, a reverse translational analysis using supervised-feature selection identified a circulating 7-miRNA signature as a predictor of population variability in CYP2B6 activity based on screening of expression of 2510 miRNAs in a multi-dimensional covariate analysis of efavirenz PK (67). The incorporation of big data and ML into the PMX pipeline to enhance the structural, population, and covariate modeling processes (Fig. 2) has also been recently proposed. For example, by leveraging RF regression and Bayesian network to identify the covariate model and harness information in external database, such a modeling paradigm can enable the obtention of an augmented population via simulations and the potential identification of informative and parsimonious QSP models (68). The value of automatizing and optimizing variables and model selections with ML has been reported in various studies (69, 70). For example, a simulation case study highlights the potential of ML-based techniques like NN and RSF to provide more accurate and robust time-to-event analysis in clinical studies over conventional approaches. Particularly, this is true when handling high-dimensional data and when the predictor variables assume nonlinear relationships in the hazard function (71). Other successful examples of ML applications outperforming traditional survival models include the integration of electronic health records from a large cohort of patients for improved predictions of patients’ mortality with RF and elastic net regression (72). The learning of data-driven associations between the longitudinal data available in primary care records and various associated risks by using a DL approach enabled the identification of covariates that are influential for different competing risks (73). Interest in combining ML with causal inference tools (e.g., inverse-probability weighting and marginal structural model) has also been reported (74).

The estimation of the optimal dose and dosing regimens recommended for a given subpopulation, planned for use in subsequent studies, and ultimately in prescribing guidance/labeling is informed by population PK modeling. This analysis is a well-established, quantitative method that can quantify and explain the variability in drug concentrations among individuals by mechanistically describing the drug PK properties (42). Some investigations were performed to compare the performance of conventional population PK modeling with NN, SVM, and tree-based ML algorithms in predicting blood concentrations at various time points (75) as well as relevant exposure metrics (76). However, given the need of interpretable models and thorough understanding of biological-physiological assumptions behind traditional population PK model formulations, the value of employing ML methods in this context is expected to be limited to situations when traditional modeling approaches are not suited. For instance, ML methods can be helpful when the drug exhibits nonlinear PK properties that cannot be easily represented by differential equations, or outcomes can inform improved trial designs and dosing regimens. An example for the latter is represented by the use of a genetic algorithm based on mechanism-based model for bacterial killing and population PK models to define optimal dosing strategies for meropenem and polymyxin B antibiotic combination therapy (77).

ML models can provide valuable insights into the importance of dosage in the real-world use of drugs in the context of a multitude of demographic and clinical factors. For example, the critical importance of attaining target metformin dosage for optimal glycemic control could be demonstrated in a large reverse translational ML analysis of administrative claims data for 12,147 commercially insured adults and Medicare Advantage beneficiaries with prediabetes or diabetes in the context of 58 demographic, laboratory, and co-morbidity-related patient-specific factors (78). This analysis used an ensemble model incorporating diverse methods (e.g., RF, gradient boosting, regression splines, discriminant analysis) to allow patient-specific prediction of 1-year glycemic control for adult patients with either diabetes or prediabetes who are newly prescribed metformin. Most analyses of this nature have tended to be cross-sectional in nature or have considered outcomes averaged over time, indicating a largely untapped opportunity for integration of ML into disease trajectory/progression models to augment quantitative estimation of predictors of optimal dosage in real-world settings. Clearly, an important enabler for longitudinal analyses to interrogate the impact of dosage on outcomes is the availability of adequately annotated clinico-pathological datasets that include sufficient temporal resolution of dosing.

As our understanding of the determinants of drug metabolism and transport processes and their regulation across patient populations become increasingly multi-dimensional, enabled by integration of endogenous biomarkers and plasma-derived nanovesicles for LBx in drug development (79) and growing knowledge of the contribution of the microbiome (80) to variability in drug disposition, ML approaches will become an indispensable part of our emerging toolkit in characterizing sources of variability in drug exposure and response.

## PERSPECTIVE

The application of AI and ML in drug discovery and development is growing. The pharmaceutical industry has already embarked on a journey of digital transformation by bridging data silos and implementing technological solutions for a more efficient use of data generated across R&D pipelines. Given its data- and question-driven strategic foundation, Translational Medicine must be ready to embrace and integrate such tools to augment conventional approaches and nurture new cross-functional collaborations that can maximize the value of data. As such, in cross-functional pharmaceutical R&D team settings, joint efforts of Clinical Pharmacology, Bioinformatics, and Biomarker Technology experts is vital to realize the promise of AI/ML-enabled Translational and Precision Medicine. The successful adoption of these methods requires (i) a clear definition of the context of use and (ii) data needs including quantity and quality requirements; and (iii) a fit-for-purpose approach thus ensuring that the application of ML is guided by sound rationale and aimed at addressing questions justifying the additional benefit of adopting these new approaches over conventional ones. Furthermore, interoperability considerations and validation, generalizability, and interpretability of models are crucial for scalability and confidence in results for the intended applications. For the latter, special attention should be made to ensure that models are interpretable and reproducible, enabling biological insights to be extracted directly or by integrating interpretable output into more mechanism-based analytical methods that are more conventionally used. In fact, ML and especially DL approaches have often been criticized for their dependency on large amounts of training data and the lack of intuition associated with the generated features. However, methods are being developed to improve the interpretability and visualization of results, thus overcoming the black box nature of some algorithms. Among these, the adoption of SHAP has greatly increased the understanding of feature importance from its local to global contribution to the ML model’s prediction. Another key aspect to consider is the generalization of the ML model to new, unseen data, still representative of the wider considered population. In this respect, as for other data-driven predictive models, online retraining of a ML model should be based on the most recent data sources. Furthermore, it is important to avoid sampling bias by ensuring a diverse training dataset leading to results that can be generalized to the entire studied population. It is also important to be aware of co-segregation among covariates, including “hidden” non-observed causal factors, that can impact interpretation of identified predictors and their relative importance. On the other hand, ML methods have shown to offer competitive advantages over conventional methods, especially when the integration and analysis of large, multi-dimensional, and heterogenous data sets is in scope. Without the need to explicitly make assumptions on the underlying data and systems relationships, such methods can provide relevant insights that can be used for hypothesis generation and be complemented by subsequent assessments in more mechanistic MID3 frameworks. A highly inter-dependent and iterative interplay across the disciplines of Quantitative Systems Pharmacology and Pharmacometrics is envisioned. Big data and advanced technologies, including but not limited to AI and ML, hold substantial promise to enable effective forward and reverse translational discovery of sources of variation in benefit-risk profiles to ultimately bring the right therapeutic solution to all patients and make precision medicine a reality.

### Section for Authors

Nadia Terranova associates her scientific curiosity and cross-disciplinary expertise to both her academic and professional career and to the several enriching collaborations she had the pleasure be involved in with female and male thought leaders in the Clinical Pharmacology and Pharmacometrics community such as L.J.B. and K.V. The natural energy and team spirit that she has been demonstrating throughout her scientific career also stem from her past experiences as a professional volleyball player. Karthik Venkatakrishnan is grateful for many fulfilling experiences from engagement with several mentors, collaborators, and mentees, including female scientists and leaders over the course of his career. He cherishes this special opportunity to collaborate with an energetic early career scientist who constantly pushes the boundaries of innovation (N.T.) and a deeply respected and distinguished leader and mentor in clinical pharmacology and translational medicine (L.J.B). Lisa Benincosa attributes the open and inclusive leadership she received during both graduate school and her professional experience. She was fortunate to have good role models including successful female models for inspiration.
